# Brief Peer-Supported Web-Based Skills Training in Affective and Interpersonal Regulation (BPS webSTAIR) for Trauma-Exposed Veterans in the Community: Randomized Controlled Trial

**DOI:** 10.2196/52130

**Published:** 2024-10-02

**Authors:** Laura E Ong, Sarah Speicher, Diana Villasenor, Jamie Kim, Adam Jacobs, Kathryn S Macia, Marylene Cloitre

**Affiliations:** 1 Department of Psychology Northern Illinois University DeKalb, IL United States; 2 National Center for Posttraumatic Stress Disorder Veterans Affairs Palo Alto Health Care System Palo Alto, CA United States; 3 Department of Psychiatry and Behavioral Sciences Stanford University Stanford, CA United States; 4 Veterans Affairs Health Systems Research Center for Innovation to Implementation Veterans Affairs Palo Alto Health Care System Palo Alto, CA United States

**Keywords:** posttraumatic stress disorder, PTSD, depression, depressive symptoms, veterans, veterans health, mHealth, mobile health, peer support, peer-to-peer, transdiagnostic, mental health, mental health services, community, emotion regulation, interpersonal regulation, mHealth program

## Abstract

**Background:**

Peer-supported mobile health (mHealth) programs hold the promise of providing a low-burden approach to increasing access to care and improving mental health. While peer support has been shown to improve engagement in care, there is limited investigation into the impact of peers on symptom outcomes. Trauma-exposed populations frequently endure co-occurring posttraumatic stress and depressive symptoms as well as difficulties in day-to-day functioning. This study evaluated the potential benefits of a peer-supported, transdiagnostic mHealth program on symptom outcomes and functioning.

**Objective:**

This randomized controlled trial tested the effectiveness of Brief Peer-Supported (BPS) web-based Skills Training in Affective and Interpersonal Regulation (webSTAIR), a 6-module transdiagnostic digital program derived from Skills Training in Affective and Interpersonal Regulation and compared to waitlist control in a community sample of veterans who screened positive for either posttraumatic stress disorder (PTSD) or depression.

**Methods:**

A total of 178 veterans were enrolled in this study using a 2:1 randomization scheme with 117 assigned to BPS webSTAIR and 61 assigned to waitlist control. PTSD and depressive symptoms as well as emotion regulation and psychosocial functioning were assessed at pretreatment, posttreatment, and 8-week follow-up time points. Mixed-effects models were used to assess change in outcome measures across time points. Exploratory analyses were conducted to determine whether the type and number of peer interactions influenced outcomes.

**Results:**

Significant interaction effects were observed for all outcomes such that participants randomized to BPS webSTAIR reported significantly greater improvement at the posttreatment time point compared to waitlist control with moderate effect sizes for PTSD (*d*=0.48), depression (*d*=0.64), emotion regulation (*d*=0.61), and functional impairment (*d*=0.61); gains were maintained at 8-week follow-up. An initial cohort of participants who were required to engage with a peer coach to progress through the modules interacted more frequently with peers but completed fewer modules compared to a later cohort for whom peer engagement was optional. Overall, those who completed more modules reported greater improvement in all outcomes.

**Conclusions:**

BPS webSTAIR was effective in improving PTSD and depression symptoms, emotion regulation, and psychosocial functioning in community veterans. Peer-supported, transdiagnostic mHealth programs may be a particularly efficient, effective, and low-burden approach to improving mental health among trauma-exposed populations. Investigation of peer-supported programs among other populations is necessary to evaluate the generalizability of the findings. Analyses comparing peer support that was required versus optional indicated that some veterans may not need or want peer support. Future research should evaluate how best to deliver peer support and for whom it is most beneficial. If successful, peer-supported tech programs may increase the Veteran Affairs workforce as well as improve veteran mental health services and outcomes.

**Trial Registration:**

ClinicalTrials.gov NCT04286165; https://clinicaltrials.gov/study/NCT04286165

## Introduction

### Background

Among US veterans, the prevalence of comorbid posttraumatic stress disorder (PTSD) and major depressive disorder is estimated to be roughly twice the rate of PTSD alone [1], and the symptoms of each of these disorders have been associated with deficits in emotion regulation [2,3] and psychosocial functioning [4,5]. Meanwhile, veterans experience numerous practical and cultural barriers to accessing evidence-based mental health treatment. These include the need to travel long distances to facilities, financial constraints, health conditions, inflexible work schedules, and caregiver responsibilities [6-8], as well as veterans’ desire to be self-reliant and the perceived stigma of receiving mental health care [9,10]. There are, in addition, systemic barriers including a shortage of mental health providers in many parts of the United States [11]. Mobile health (mHealth) programs, including web-based and app programs, address these difficulties and have been found to be effective in the treatment of symptoms of PTSD and depression [12-15]. However, it has been shown that mHealth programs with human support consistently provide greater benefits than entirely self-guided programs [16,17]. Given the burdens on clinicians and other licensed professionals in effectively serving patient needs, the introduction of peer-supported mHealth programs holds the promise of reducing the burden on the health care system while maintaining and potentially increasing access to care and supporting patients in obtaining optimal outcomes.

Peer specialists are persons with lived experience of recovery from mental health difficulties who are trained to support patients with similar problems in engaging in and completing mental health programs [18]. Peer specialists bring particular strengths to their roles, including their ability to develop strong rapports with participants and reduce stigma [19,20]. Literature reviews have indicated that the presence of peers increases engagement in treatment across persons with a variety of mental health difficulties and disorders, treatment modalities, and delivery strategies [18,21]. In particular, the use of peer support in mHealth has been found to increase engagement among veterans with unmet mental health needs [22]. However, randomized controlled trials assessing the impact of peers on symptom change are relatively few in number [23] including for mHealth interventions [21].

Studies integrating peer specialists into Veteran Affairs (VA) mHealth programs have strongly supported their acceptability and feasibility [22,24-26]. Randomized controlled trials of peer-supported, web-based programs have reported moderate to modest symptom change outcomes on PTSD and depression when compared to waitlist [27] and found that utilization of peer support was associated with greater reductions in depression when compared to self-directed program usage [22]. Additional trials are needed to assess mental health outcomes, particularly symptom change and psychosocial functioning to better understand the optimal contribution of peer-supported mHealth programs to mental health care. This study evaluates a peer-supported, transdiagnostic mHealth program for trauma-exposed veterans. Given the frequency of comorbid symptomatology among trauma-exposed populations [1,28], a transdiagnostic approach, if successful, provides the opportunity for broad use across various trauma-exposed populations with diverse needs. Moreover, different types of symptoms share issues with emotion regulation and psychosocial functioning, making these difficulties important treatment targets.

The program evaluated, web-based Skills Training in Affective and Interpersonal Regulation (webSTAIR), is a web-based program adapted from Skills Training in Affective and Interpersonal Regulation (STAIR), a manualized evidence-supported cognitive behavioral intervention for trauma-exposed persons with symptoms of PTSD or depression that focuses on improving psychosocial functioning by building emotion regulation and interpersonal skills. Investigations of 10-12 session STAIR have reported large effect sizes in the reduction of PTSD and depression as well as in emotion regulation and psychosocial impairment [29,30]. Similar outcomes have been obtained in a briefer 6-session version, including in a randomized controlled trial in primary care [31] and in an open trial delivered by peers in a low-income, primary care community service [32]. In parallel to the STAIR studies, investigations of webSTAIR assessed a 10-module version with weekly coaching sessions from licensed mental health providers. These studies have included 2 open trials [33,34] and 1 comparison trial [35]. The open trials found significant improvements with moderate to large effect sizes for PTSD, depression, emotion regulation difficulties, and psychosocial impairment, and the comparison study, with a noninferiority design, found that reducing coaching support to biweekly (5 coaching sessions) from weekly (10 coaching sessions) resulted in noninferior benefits on all outcomes.

Given the evidence supporting the success of brief, peer-supported STAIR in reducing a range of symptoms, along with growing support for the effectiveness of web-based mental health programs, a 6-session peer-supported version of webSTAIR was developed. This abbreviated version of the webSTAIR protocol can be completed in a shorter time frame than the 10-session webSTAIR and integrates support from trained veteran peers at the end of each module. The potential benefits of this program, if successful, is the availability of a low-burden, brief mHealth program that is effective in reducing PTSD and depression symptoms as well as improving emotion regulation and psychosocial functioning.

### Objectives

This study aimed to assess the benefits of Brief Peer-Supported (BPS) webSTAIR compared to a waitlist control condition among US veterans with symptoms of PTSD, depression, or both. We hypothesized that participants assigned to the BPS webSTAIR condition would experience significantly greater improvement in PTSD symptoms and depressive symptoms (primary outcomes) as well as in emotion regulation and psychosocial functioning (secondary outcomes) compared to the waitlist, and that these gains would be maintained at an 8-week follow-up. Exploratory analyses were conducted to determine whether the number and type of peer interactions influenced outcomes. This study was funded by the National Center for PTSD Dissemination and Training Division within the VA Palo Alto Health Care System.

## Methods

### Procedures

Candidates for study participation were recruited via social media advertisements and directed to complete a short set of online screening questions. Following the screening, study candidates were contacted via phone and scheduled for a 30- to 60-minute phone assessment with this study’s coordinator to assess inclusion and exclusion criteria, receive an explanation of the program, and complete (verbal) informed consent. If eligible for this study, the participants were provided access to the program. Randomization was allocated at a 2:1 ratio (webSTAIR to waitlist) in blocks of 12 and was computer generated via an algorithm developed by staff otherwise not involved in this study. Participant randomization assignment was indicated upon opening the program URL. Participants in the BPS webSTAIR condition received instructions to start the program within 2 weeks following the assessment and to complete the 6-module program within 10 weeks. Posttreatment assessment (10-week mark) and an 8-week follow-up were conducted via phone by study staff who were unaware of the condition and assessment period. Participants in the waitlist condition were given the information that they would have access to the webSTAIR program in 10 weeks and completed assessments at the pretreatment and posttreatment time points (10-week mark). Waitlist participants were then given access to webSTAIR. Recruitment began on October 16, 2020, and follow-up ended on September 28, 2022. This study is registered at ClinicalTrials.gov (NCT04286165).

### Participants

Persons were eligible if they were veteran or military personnel; aged older than 21 years; reported experiencing at least 1 traumatic event; reported symptoms of PTSD or depression, or both, as indicated by a score of ≥3 on the Primary Care Posttraumatic Stress Disorder Screen for *Diagnostic and Statistical Manual of Mental Disorders, Fifth Edition* [36] or ≥2 the Patient Health Questionnaire-2 item (PHQ-2) [37]; were able to read and write in English; had a PC and stable internet connection; and resided in the United States. Exclusion criteria were determined during the phone assessment and were (1) the presence of significant suicidality as indicated by the presence of a plan and means, (2) the presence of cognitive difficulties or active psychosis that would indicate a low likelihood of benefiting from treatment, and (3) current participation in trauma-focused treatment.

### Measures

#### Overview

The pretreatment assessment included a series of sociodemographic questions and the Life Events Checklist (LEC) [38], which in this study was used to measure only those potentially traumatic events that directly happened to the person, in addition to the measures below. The posttreatment assessment was scheduled for week 10 of this study and follow-up at 8 weeks after treatment.

#### PTSD Symptoms

PTSD symptoms were measured using the Posttraumatic Stress Disorder Checklist for *Diagnostic and Statistical Manual of Mental Disorders, Fifth Edition* (PCL-5) [39]. This 20-item self-report measure asks participants to indicate how much they have been bothered by each symptom in the past month, on a scale from 0 (“not at all”) to 4 (“extremely”). A total score is calculated by summing all items, where higher scores correspond to greater PTSD symptom severity. A cutoff score of 31-33 points or more has been established as indicating probable PTSD [40]. The PCL-5 has demonstrated excellent internal consistency, good test-retest reliability, and excellent convergent validity among veterans [40]. The baseline Cronbach α of the PCL-5 in the current sample was 0.92.

#### Depressive Symptoms

Depressive symptoms were measured using the Patient Health Questionnaire-8 item (PHQ-8) [41]. The PHQ-8 is a self-report measure that comprises the first 8 items of the Patient Health Questionnaire-9 item (PHQ-9). It asks participants to rate how often they have been bothered by each problem over the past month, on a scale from 0 (“not at all”) to 3 (“nearly every day”). A total score is calculated by summing all items, where higher scores correspond to greater depressive symptom severity. The PHQ-8 has demonstrated good internal consistency and convergent validity among both psychiatric outpatients and the general population [41,42]. The baseline Cronbach α of the PHQ-8 in the current sample was 0.83.

#### Emotion Regulation

Emotion regulation was measured using the Difficulties in Emotion Regulation Scale-16 item (DERS-16) [43]. This 16-item self-report measure asks participants to rate how often each of a series of statements applies to them on a scale from 1 (“almost never”) to 5 (“almost always”). A total score is calculated by summing all items, where higher scores correspond to greater difficulties with emotion regulation. The DERS-16 has demonstrated excellent internal consistency, good test-retest reliability, and good convergent and discriminant validity [44]. The baseline Cronbach α of the DERS-16 in the current sample was 0.93.

#### Psychosocial Functioning

Psychosocial functioning was measured using the Work and Social Adjustment Scale (WSAS) [45]. This 5-item self-report measure asks participants to rate their degree of impairment in carrying out each of a series of activities on a scale from 0 (“not at all”) to 8 (“very severely”). A total score is calculated by summing all items, where higher scores correspond to greater functional impairment. The WSAS has demonstrated strong psychometric properties among psychiatric samples [45,46]. The baseline Cronbach α of the WSAS in the current sample was 0.82.

#### Engagement and Completion Data

Metadata on chat sessions, including the number of messages exchanged with a peer coach, was collected at the participant level. Measures of program engagement within the BPS webSTAIR condition included the number of modules completed, the total number of messages exchanged, and the average number of messages exchanged per module.

### Treatment

The BPS webSTAIR intervention is a shortened, 6-module version of webSTAIR adapted from brief STAIR [31]. The 6 modules of BPS webSTAIR focus on emotional awareness; emotion management through the body, thought, and behavior channels; distress tolerance; and self-compassion. The program integrates text, audio, and video delivery of psychoeducation with interactive exercises and worksheets to help patients understand and internalize the concepts introduced.

At the end of each module, participants were prompted to complete a chat session with a veteran peer coach to help clarify program content and answer any questions they might have about how to implement the skills presented in the module. Interaction with the peer occurred via secure instant messaging on the program platform. Participants could message peers as often as they liked while they were on the platform. It was not expected that the participant would receive a response from the same peer. The peer program was committed to responding to text messages within 24 hours. Due to changes in the availability of veteran peer coaches over this study’s period, 3 distinct cohorts were identified based on participant start date. In cohort 1 (from October 16, 2020, to July 9, 2021), coaches were available for 24/7 support, and participants were required to complete a chat session with a coach at the end of each module to move forward to the next module. In cohort 2 (from July 10, 2021, to November 22, 2021), coach availability was reduced to 8 hours a week, with 4 hours at the beginning of the day; 8:30 AM-12:30 PM) and 4 hours at the end of the day and into the evening (4:30 PM). In cohort 3 (from November 23, 2021, to July 20, 2022), coach availability remained reduced, but participants were able to independently skip any chat sessions they chose. For all 3 cohorts, the research coordinator was available as backup for logistical and content support Monday through Friday by phone call or secure text message during an 8-hour workday (8 AM to 4 PM Pacific) with responses completed within 24 hours or less.

### Veteran Peer Coaches

All veteran peer coaches were VA-certified peer counselors, which includes a working knowledge of VA guidelines for assessing suicidal risk and referral to the veteran suicide hotline. In addition, for this project, the peers received training from the developers of the web-based platform about navigation of the system (Vets Prevail) and received a manual and a workshop consisting of two 2-hour meetings taught by webSTAIR staff (MC and SS) which described the interventions and provided suggestions about how to connect the program material to ongoing stressors in the life of the veteran client and to recommend the practice of skills between sessions.

### Statistical Analysis Plan

Analyses were conducted using R (version 4.2.2; R Foundation for Statistical Computing). All participants who met eligibility requirements and completed the pretreatment assessment were included in the final dataset, regardless of whether they completed the program or participated in subsequent assessments. Consequently, all analyses were conducted using the intention-to-treat (ITT) sample of randomized participants. Categorical pretreatment demographic variables and responses to individual LEC items were compared between the treatment and control groups using chi-squared tests of independence. The mean LEC scores for the 2 groups were compared using independent samples 2-tailed *t* tests. Rates of missingness were 0% (0/178) at baseline, 38.2% (68/178) at posttreatment assessment with 47% (55/117) missing for webSTAIR and 21% (13/61) missing for waitlist, and 41.9% (49/117) at follow-up (webSTAIR condition only). Missing data were addressed using multiple imputation. Joint multiple imputation of missing values at posttreatment and follow-up assessments was carried out using the *panImpute* function in package *mitml*. Due to (1) some of the presently reported analyses being specific to webSTAIR participants, while other analyses were relevant to the full sample, (2) differences in the assessment schedule between the treatment and control conditions (ie, only webSTAIR participants were assessed at follow-up), (3) the unbalanced randomization design, and (4) differences in rates of missingness between conditions, multiple imputation procedures were carried out separately for the data that would be used to examine within-group change for webSTAIR participants across the 3 time points and between-group differences in change from pre- to posttreatment assessments. In other words, the between-group imputed datasets consisted of baseline and posttreatment data for all participants, whereas the within-group imputed datasets included baseline, posttreatment, and follow-up data for only the participants randomized to webSTAIR. In each case, 100 datasets were generated with missing values imputed. Before imputing the data, chi-square tests and 2-tailed *t* tests were used to identify predictors of missingness and baseline characteristics related to nonmissing values on outcome variables. The missing data correlates identified from these preliminary analyses (employment status, education level, service era, and gender) were included as auxiliary variables in the imputation models. To ensure that any interactions between time and condition were appropriately preserved in the between-group imputed datasets, this interaction term was included as a predictor in the between-group imputation model. The results were pooled across imputed datasets using the test estimates function in the *mitml* package.

For the main analyses of our primary and secondary outcomes, linear mixed-effects models were fitted using restricted maximum likelihood estimation with the *lmer* function in the package *lme4*. These models included main effects for time and condition, a time-by-condition interaction, as well as fixed effects to adjust for potential differences between the cohorts that may have resulted from changes in peer accessibility and chat requirements during this study. To control for potential cohort effects, 2 dummy variables were created to identify the participants in cohorts 2 and 3, with cohort 1 as the reference group. A random intercept was included in all models to account for repeated measurements within participants. The time by condition interaction term provided a test of the difference in change from pre- to posttreatment assessments between the webSTAIR and waitlist conditions. Cohen *d* between-group effect sizes for each measure were calculated by dividing the estimated interaction term by the SD of pretreatment scores in the total sample. Similar models were constructed to measure within-group change among webSTAIR participants across all 3 time points. The time predictor in these models was specified so that posttreatment assessment was the reference category, which enabled us to evaluate the magnitude and statistical significance of changes that occurred from pre- to posttreatment assessments, as well as whether any improvements during treatment were retained during the follow-up period (ie, as indicated by a nonsignificant estimate for change between the posttreatment and follow-up assessments). In the within-group analyses, Cohen *d* effect sizes for change from pre- to posttreatment assessment and from posttreatment to follow-up assessment in the BPS webSTAIR group were calculated by dividing the estimated coefficients for time by the SD of pretreatment scores for the BPS webSTAIR condition.

To evaluate the impact of program completion on outcomes, module completion was categorized as “none” (did not complete any web-based content, including the welcome module), “some” (completed the welcome module and no more than module 2), or “moderate to complete” (completed module 3 or higher). Within each completion group, linear mixed-effects models were constructed for each outcome measure to evaluate within-group change across time. Time predictor dummies were specified such that posttreatment assessment was the reference category, and a random intercept was included in each model. Cohen *d* effect sizes for pre- to posttreatment change within each completion group were calculated by dividing the estimated coefficient for time by the SD of pretreatment scores for BPS webSTAIR participants in the respective completion group (eg, the SD of pretreatment PCL-5 scores among BPS webSTAIR participants in the “moderate to complete” group).

Exploratory analyses were conducted to better understand the impact of the type (required versus optional) and number of peer chat sessions in the different cohorts. For each participant, we calculated the total number of chat messages exchanged with a peer support coach and the average number of chat messages exchanged per module of the program completed. Descriptive statistics for chat messages were examined by cohort. Comparisons were drawn between cohort 1, in which chat sessions were required to advance to the next module, and cohort 3, in which chat sessions were not required for module advancement, to assess whether chat engagement and completion rates differed in these 2 contexts. We did not include cohort 2 in these analyses, given that this was a transitional phase when participants’ experiences with the chat feature may have varied in ways that are not quantifiable (eg, experienced highly variable wait times in connecting with a peer).

Finally, the relationship of chat messages with treatment outcomes was explored by fitting linear regression models that included average messages exchanged per module and pretreatment scores for the outcome as predictors of pre- to posttreatment change in the outcome. Due to differences between cohorts in the availability of peer coaches and program instructions for module completion, the relationships between messages exchanged per module and changes in outcomes could not be interpreted in the same way across cohorts. Given these differences in context, separate models were estimated for each cohort so that relationships between chat engagement and outcomes could be interpreted according to the specific circumstances of each cohort. Additionally, differences in the number of modules completed by participants were taken into account by examining the relationships between messages exchanged *per module* (vs total messages exchanged) and change in outcomes.

### Ethical Considerations

Institutional review board approval for the study was obtained from Stanford University (Protocol 51076). All participants provided verbal informed consent. Participants were paid US $40 for the pretreatment assessment and US $60 for the posttreatment and follow-up assessments. Data were collected online and stored in a secure VA-approved environment.

## Results

**Figure 1:**
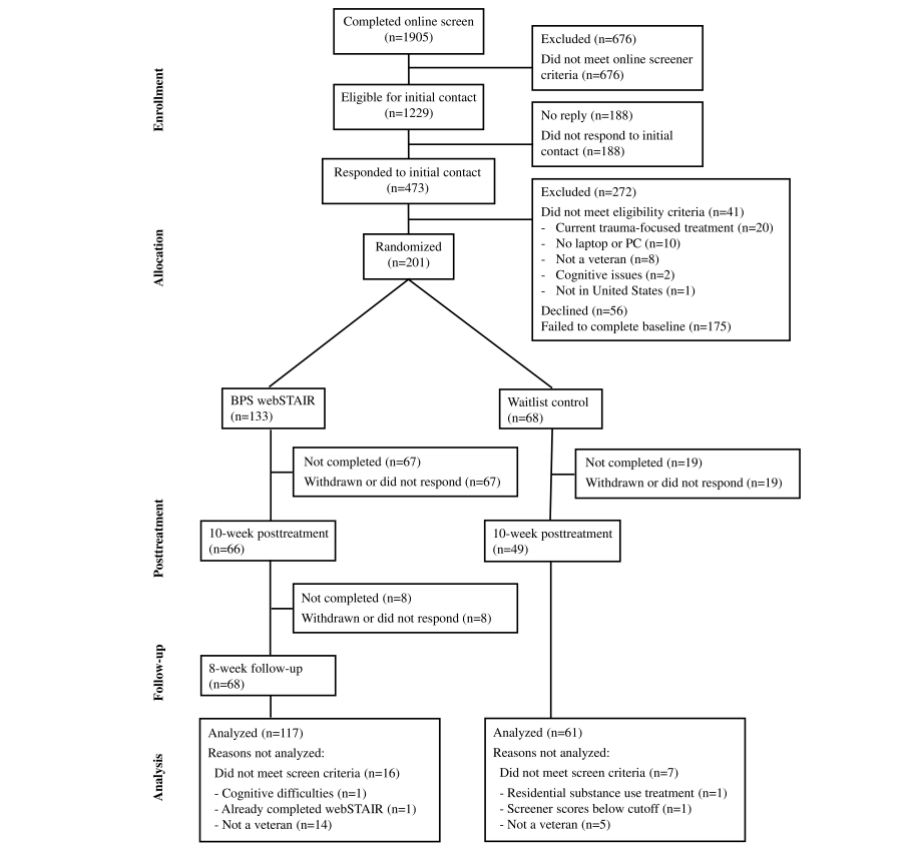
CONSORT (Consolidated Standards of Reporting Trials) diagram.

A total of 178 eligible veterans were enrolled in this study using a 2:1 randomization scheme with 117 assigned to BPS webSTAIR and 61 assigned to the waitlist control. As indicated in the CONSORT chart (Figure 1), 201 participants were initially enrolled but 23 participants (16 in webSTAIR and 7 on waitlist) were excluded from this study predominantly due to low credibility regarding their veteran status (eg, did not know their rank or were uncertain about which branch of the armed forces they served in). Sample sociodemographic characteristics are shown in Table 1. Similar to other veteran samples, most participants were male (125/178, 70.2%). Roughly two-thirds (120/178, 67.4%) were White and the average age was 48.08 (SD 9.04) years. A total of 57.9% (103/178) were approved for VA service connection status for PTSD (at any disability rating), 27.0% (48/178) had never applied, 8.4% (15/178) had applied or had an application under review, and 6.7% (12/178) had an application denied. A total of 32.0% (57/178) were currently engaged in other (non–trauma-focused) counseling, and 6.7% (12/178) had received evidence-based treatment for PTSD or related conditions within the past year. No significant differences by condition were found for any sociodemographic item (all *P*>.05). At the pretreatment assessment, the 2 conditions did not significantly differ on any outcome measures except for the PHQ-8 (*P*=.03).

**Table 1:** Sociodemographic and clinical characteristics of participants at baseline.

Baseline characteristic			BPS^a^ webSTAIR^b^ (n=117)		Waitlist (n=61)		*P* value	
**Age^c^ (years), mean (SD)**			47.66 (9.31)		48.90 (8.49)		.38	
**Race or ethnic background, n (%)**								.49
	White	80 (68.4)		40 (66)				
	Black or African American	17 (14.5)		9 (15)				
	Hispanic, Latino/a, or Mexican	9 (7.7)		7 (12)				
	American Indian or Alaskan Native	4 (3.4)		3 (5)				
	Native Hawaiian or Pacific Islander	2 (1.7)		1 (1.6)				
	Asian	0 (0)		1 (1.6)				
	Other	5 (4.3)		0 (0)				
**Self-identified gender, n (%)**								.32
	Male	81 (69.2)		44 (72)				
	Female	36 (30.8)		16 (26)				
	Transgender	0 (0)		1 (2)				
**Lifetime trauma (LEC^d^), mean (SD)**			10.92 (2.7)		10.84 (3)		.84	
**Baseline outcomes, mean (SD)**								
	PCL-5^e^	48.41 (14.55)		43.93 (16.84)		.08		
	PHQ-8^f^	15.41 (5.23)		13.48 (5.66)		.03		
	DERS-16^g^	52.44 (13.95)		48.18 (15.26)		.07		
	WSAS^h^	27.05 (8.70)		25.25 (9.14)		.21		

^a^BPS: Brief Peer-Supported.

^b^webSTAIR: web-based Skills Training in Affective and Interpersonal Regulation.

^c^Age data were unavailable for 1 participant in the webSTAIR condition and 2 participants in the waitlist condition.

^d^LEC: Life Events Checklist.

^e^PCL-5: Posttraumatic Stress Disorder Checklist for Diagnostic and Statistical Manual of Mental Disorders, Fifth Edition

^f^PHQ-8: Patient Health Questionnaire-8 item.

^g^DERS-16: Difficulties in Emotion Regulation Scale-16 item.

^h^WSAS: Work and Social Adjustment Scale.

Table 2 presents the imputed means and SDs of scores on outcome measures by condition across time points, along with the model-implied Cohen *d* effect size measures for between-group differences in change and overall change within the webSTAIR group, derived from the linear mixed-effects model results. Significant condition×treatment interaction effects were observed for the PCL-5 (*P*=.02), PHQ-8 (*P*=.01), DERS-16 (*P*=.003), and WSAS (*P*=.045), indicating that webSTAIR participants experienced significantly greater improvements from pre- to posttreatment assessment than waitlist participants. The results of the mixed-effects models examining change in outcomes across the 3 time points at which webSTAIR participants were assessed similarly indicated that webSTAIR participants experienced significant improvements on all measures between pre- and posttreatment assessment: PCL-5 (*P*=.001), PHQ-8 (*P*<.001), DERS-16 (*P*<.001), and WSAS (*P*<.001). Improvements experienced during treatment on all outcome measures were maintained through follow-up with no significant changes observed from posttreatment to follow-up assessment for any of the outcome measures (all *P*>.05). Male veterans did not differ from veterans who identified as other genders on any of the outcome measures at posttreatment (PCL-5: *P=*.50; PHQ-8: *P=*.31; DERS-16: *P=*.22; WSAS: *P=*.42) or follow-up assessment (PCL-5: *P=*.47; PHQ-8: *P=*.18; DERS-16: *P=*.21; WSAS; *P=*.15).

**Table 2:** Imputed means, SD, and Cohen d effect sizes of outcomes^a^.

Measure and condition		Pretreatment assessment	Posttreatment assessment		Follow-up assessment	Time (pre- to posttreatment assessment) × condition interaction				Pre- to posttreatment change for BPS^b^ webSTAIR^c^					Posttreatment to FU^d^ change for BPS webSTAIR			
						Cohen *d* (95% CI)		*P* value		Cohen *d* (95% CI)		*P* value		Cohen *d* (95% CI)			*P* value	
**PCL-5^e^**							–0.43 (–0.78 to –0.07)		.02		–0.48 (–0.76 to –0.21)		.001			0.05 (–0.23 to 0.32)		.75
	BPS webSTAIR	48.41 (14.55)	41.28 (17.56)		42.09 (18.28)													
	Waitlist	43.93 (16.84)	43.39 (17.86)		—^f^													
**PHQ-8^g^**							–0.51 (–0.90 to –0.12)		.01		–0.64 (–0.87 to –0.40)		<.001			0.12 (–0.15 to 0.39)		.38
	BPS webSTAIR	15.41 (5.23)	12.05 (5.77)		12.71 (6.13)													
	Waitlist	13.48 (5.66)	12.88 (5.77)		—													
**DERS-16^h^**							–0.60 (–0.99 to –0.21		.003		–0.61 (–0.85 to –0.36)		<.001			0.07 (–0.19 to 0.34)		.60
	BPS webSTAIR	52.44 (13.95)	43.04 (15.10)		44.95 (15.97)													
	Waitlist	48.18 (15.26)	47.50 (14.82)		—													
**WSAS^i^**							–0.38 (–0.74 to –0.01)		.045		–0.61 (–0.87 to –0.35)		<.001			0.13 (–0.12 to 0.38)		.32
	BPS webSTAIR	27.05 (8.70)	21.74 (10.44)		22.83 (10.72)													
	Waitlist	25.25 (9.14)	23.27 (10.40)		—													

^a^All models controlled for potential cohort effects.

^b^BPS: Brief Peer-Supported.

^c^webSTAIR: web-based Skills Training in Affective and Interpersonal Regulation.

^d^FU: follow-up.

^e^PCL-5: Posttraumatic Stress Disorder Checklist for Diagnostic and Statistical Manual of Mental Disorders, Fifth Edition.

^f^Not applicable.

^g^PHQ-8: Patient Health Questionnaire-8 item.

^h^DERS-16: Difficulties in Emotion Regulation Scale-16 item.

^i^WSAS: Work and Social Adjustment Scale.

Table 3 shows the estimated coefficients and effect sizes for pre- to posttreatment change in the outcome measures by module completion group in the BPS webSTAIR condition. Participants who completed no modules (n=25; coded as “None”) did not experience a significant change in PCL-5 score from pretreatment to posttreatment assessment (estimate=–1.88, SE 4.72; *P*=.69; *d*=–0.13). Those who completed at least the welcome module but did not move beyond the second full module of BPS webSTAIR (n=48; coded as “Some”) experienced a mean PCL-5 total score reduction of 6.77 (SE 3.16; *P*=.03; *d*=–0.50). Participants who completed 3 or more (out of 6 total) modules of BPS webSTAIR (n=44; coded as “Moderate to Complete”), experienced an estimated PCL-5 total score reduction of 10.13 (SE 2.49) points from pretreatment to posttreatment assessment (*P*<.001; *d*=–0.64). The PHQ-8, DERS-16, and WSAS followed a similar pattern, wherein significant pre- to posttreatment improvements were observed for the “some” and “moderate to complete” groups, but not the “none” group.

**Table 3:** Results from mixed models examining pre- to posttreatment changes in outcome by completion group.

Measure	Module completion category								
	None (n=25)^a^			Some (n=48)^b^			Moderate to complete (n=44)^c^		
	Estimate (SE)	*P* value	Cohen *d* (95% CI)	Estimate (SE)	*P* value	Cohen *d* (95% CI)	Estimate (SE)	*P* value	Cohen *d* (95% CI)
PCL-5^d^	–1.88 (4.72)	.69	–0.13 (–0.79 to 0.53)	–6.77 (3.16)	.03	–0.50 (–0.95 to –0.04)	–10.13 (2.49)	<.001	–0.64 (–0.95 to –0.33)
PHQ-8^e^	–1.15 (1.46)	.43	–0.23 (–0.82 to 0.35)	–3.09 (0.96)	.001	–0.68 (–1.09 to –0.27)	–4.82 (0.82)	<.001	–0.81 (–1.08 to –0.54)
DERS-16^f^	–2.18 (4.26)	.61	–0.13 (–0.65 to 0.38)	–8.44 (2.69)	.002	–0.70 (–1.13 to –0.26)	–12.16 (2.07)	<.001	–0.83 (–1.11 to –0.56)
WSAS^g^	–2.33 (2.62)	.37	–0.25 (–0.79 to 0.30)	–5.04 (1.74)	.004	–0.64 (–1.07 to –0.20)	–7.31 (1.32)	<.001	–0.81 (–1.10 to –0.52)

^a^Completed no modules.

^b^Completed through the welcome module, module 1, or module 2.

^c^Completed through module 3 or beyond.

^d^PCL-5: Posttraumatic Stress Disorder Checklist for Diagnostic and Statistical Manual of Mental Disorders, Fifth Edition.

^e^PHQ-8: Patient Health Questionnaire-8 item.

^f^DERS-16: Difficulties in Emotion Regulation Scale-16 item.

^g^WSAS: Work and Social Adjustment Scale.

Chat usage data are displayed for all participants in the BPS webSTAIR condition and by cohort in Table 4. Participants in cohort 1 completed a significantly higher number of chat sessions compared with participants in cohort 3 (*P*=.004). The average number of messages exchanged per module was 11.92 (SD 11.35) in cohort 1 and 4.44 (SD 9.24) in cohort 3 (*P*=.004), and participants in cohort 3 were significantly more likely to exchange no messages at all (*χ*^2^_1_=10.00, N=71, *P*=.002). However, participants in cohort 3 completed more modules, an average of 4.05 (SD 2.75) modules (including the welcome module) compared to an average of 2.45 (SD 2.68) modules completed in cohort 1 (t_65.4_=–2.47, *P*=.02). Additionally, 16 (40%) of the 40 participants in cohort 3 completed the program compared with only 6 (19%) of the 31 participants in cohort 1 (*χ*^2^_1_=3.48, N=71, *P*=.06). Regarding the relationships of chat usage with outcomes, the number of messages exchanged per module in cohort 1 was not significantly associated with pre- to posttreatment change in any of this study’s outcomes (PCL-5: *P*=.87; PHQ-8: *P*=.37; DERS-16: *P*=.84; WSAS: *P*=.49). The results were similar for cohort 3 in that the number of messages exchanged per module was not significantly associated with pre- to posttreatment change in any outcome (PCL-5: *P*=.89; PHQ-8: *P*=.67; DERS-16: *P*=.82; WSAS: *P*=.66).

**Table 4:** Peer chat usage metrics by cohort.

	Cohort 1 (n=31)	Cohort 2 (n=46)	Cohort 3 (n=40)	All cohorts (N=117)
No messages exchanged, n (%)	10 (32)	17 (37)	28 (70)	55 (47)
Total messages exchanged, mean (SD)	48.87 (62.24)	21.46 (36.09)	23.58 (53.04)	29.44 (50.90)
Messages exchanged per module^a^, mean (SD)	11.92 (11.35)	5.97 (7.79)	4.44 (9.24)	7.02 (9.73)
No modules completed, n (%)	11 (36)	13 (28)	1 (3)	25 (21.4)
Modules completed^a^, mean (SD)	2.45 (2.68)	2.43 (2.53)	4.05 (2.75)	2.99 (2.73)
Completed program, n (%)	6 (19)	8 (17)	16 (40)	30 (25.6)

^a^BPS webSTAIR (Brief Peer-Supported web-based Skills Training in Affective and Interpersonal Regulation) includes a welcome module and 6 intervention modules. Module completion is presented on a scale of 0 to 7, where 7 indicates completion of the welcome module and 6 intervention modules.

## Discussion

### Principal Findings

The results of this study indicated that as compared to the waitlist, peer-supported webSTAIR is effective in improving symptoms of PTSD and depression, as well as emotion regulation and psychosocial functioning, with gains maintained at 8-week follow-up. The within-group pre- to posttreatment effect sizes for the webSTAIR participants ranged from 0.48 to 0.64, somewhat larger than those found for other peer-supported mHealth programs [18,21]. BPS webSTAIR offers a convenient and potentially more scalable option for veterans who have limited time available, lack access to a mental health provider, or prefer to work through the content in a self-guided manner.

The outcome varied depending on the number of modules completed. Among those who completed 3 or more modules, the mean reduction in PCL-5 score was over 10 points, and for the PHQ-8, it was nearly 5 points, reductions that are typically considered clinically meaningful [47,48]. Furthermore, our findings suggest that even partial completion of BPS webSTAIR (completing at least the welcome module but not past the second module) is associated with improved PTSD and depressive symptoms, emotion regulation, and psychosocial functioning relative to the waitlist. Therefore, the program may be of some benefit to patients who are unable to fully complete it due to low functional status or other demands on their time.

Overall, only about half (62/117, 53.0%) of the veterans engaged with peers, with the highest level of nonengagement (28/40, 70%) occurring in cohort 3. Analyses of chat data may provide insights about the role of peer engagement in mHealth. Based on our analyses of chat data from cohort 1, requiring a peer chat to move to the next module of the program was associated with a greater number of chat sessions and more messages exchanged, per module and in total. However, it did not contribute to program completion. In fact, the average number of modules completed in cohort 1 and the proportion of program completers was significantly lower than that in cohort 3. The introduction of a required engagement with the peer was intended to prompt the participants into peer engagement that otherwise might not have happened and provide an experience of contact that would lead to more engagement with the program and greater completion rates; however, this did not happen. Rather, the higher module completion rate within cohort 3 suggests that participants were motivated to work through the content even without support. Similar to this study, Possemato and colleagues [22] reported that 55% of veterans provided with the option to engage with a peer chose not to and suggested that some veterans might not need or want peer support. We speculate that a flexible, personalized approach to peer use leads to better program use and potentially better outcomes. The analyses indicated that while greater module completion was associated with the cohort with flexible peer contact, and increasing module completion was associated with a better outcome, the number of contacts (peer chats) was not associated with a better outcome in either cohort. This may be due to the potential moderating effects of patient preference regarding the use of peer support. For example, only patients who need and want peer support may benefit from it while others might do equally well or better without it. It would be valuable to identify the baseline characteristics of clients who might substantially benefit from peer support versus those who will not. In addition, it may be that clients who will be helped by peer support will experience greater benefits if the peer is proactive in reaching out to the client. In qualitative interviews, mental health coaches have pointed to the value of including proactive contact initiated by coaches rather than placing the onus on participants to engage [49]. In this study, the peers were available to the veterans to chat, but the veterans initiated the contact. Future efforts to enhance the impact of peers may involve modification regarding how proactive they are.

### Limitations

This study has limitations. First, we relied fully on self-report measures administered by phone. Second, for ethical reasons, we did not maintain the waitlist group during the follow-up period and so were unable to conduct between-group analyses of change from pretreatment to follow-up. Third, this study design did not intentionally include an implementation variation regarding required versus optional engagement peers. As a result, the cohort analyses are post hoc and we have limited information about the veterans’ experience. We do not know, for example, the average wait time for a chat, the range of wait time, or the frequency of contact with the research coordinator. Differences in these factors across the cohorts could have contributed to the observed differences in retention rates. Furthermore, chat transcripts were not accessible and thus we were limited in our ability to explain why more chat messages were not associated with better outcomes. It could be that the conversations were routine and pragmatic (eg, resolving technical issues) versus meaningful guidance about the content of webSTAIR. Lastly, participants were veterans recruited via social media advertisements which may limit generalization of findings beyond for veterans or persons who do not interact with social media.

### Conclusions

This study adds to existing literature on mHealth by demonstrating that a brief, transdiagnostic, peer-supported, web-based program can provide moderate to large improvements in PTSD and depression symptoms as well as in emotion regulation and psychosocial functioning. Given the potential of peer-supported mHealth programs to expand access to evidence-based care for symptoms of PTSD and depression, future research should evaluate how best to deliver peer support, how much to deliver, and for whom the support benefits most. Future studies may also explore alternative formats for the delivery of peer support; for instance, proactive outreach from peer coaches or supplementary channels of communication (eg, phone contacts) to increase flexibility in ways in which a peer relationship can be built. The particular strengths of peer support for specific and varied aspects of mental health care such as treatment engagement, program completion, and symptom reduction need to be determined to develop maximally efficient and effective mental health services.

## Appendix

Multimedia Appendix 1 CONSORT (Consolidated Standards of Reporting Trials) checklist.

## Abbreviations

BPS: Brief Peer-Supported
DERS-16: Difficulties in Emotion Regulation Scale-16 item
ITT: intention-to-treat
LEC: Life Events Checklist
mHealth: mobile health
PCL-5: Posttraumatic Stress Disorder Checklist for Diagnostic and Statistical Manual of Mental Disorders, Fifth Edition
PHQ-2: Patient Health Questionnaire-2 item
PHQ-8: Patient Health Questionnaire-8 item
PHQ-9: Patient Health Questionnaire-9 item
PTSD: posttraumatic stress disorder
STAIR: Skills Training in Affective and Interpersonal Regulation
VA: Veteran Affairs
webSTAIR: web-based Skills Training in Affective and Interpersonal Regulation
WSAS: Work and Social Adjustment Scale
