# Supplementary material for: Brief Peer-Supported Web-Based Skills Training in Affective and Interpersonal Regulation (BPS webSTAIR) for Trauma-Exposed Veterans in the Community: Randomized Controlled Trial
Source: J Med Internet Res. 2024 Oct 2;26:e52130. doi: 10.2196/52130 (PMC11483263; doi:10.2196/52130)
Supplement: Multimedia Appendix 1 [file jmir_v26i1e52130_app1.pdf]

CONSORT-EHEALTH Checklist V1.6.2 Report	Manuscript Number	52130
**(based on CONSORT-EHEALTH V1.6), available at [http://tinyurl.com/consort-ehealth-v1-6].**		
**Date completed**		
**by**		
Jamie Kim		
Brief Peer-Supported webSTAIR for Trauma-Exposed Veterans in the Community: Randomized Controlled Trial		
TITLE		
**1a-i) Identify the mode of delivery in the title**		
webSTAIR - tested web-based version of mental health program called STAIR		
**1a-ii) Non-web-based components or important co-interventions in title**		
Brief Peer-Supported - Veteran peers were coaches in this program		
**1a-iii) Primary condition or target group in the title**		
Trauma-Exposed Veterans in the Community - face to face program administered to community sample of Veterans who screened positive for either PTSD and/or depression		
ABSTRACT		
**1b-i) Key features/functionalities/components of the intervention and comparator in the METHODS section of the ABSTRACT**		
"This randomized controlled trial tested the effectiveness of Brief Peer-Supported (BPS) webSTAIR, an abbreviated transdiagnostic online program derived from STAIR (skills training in affective and interpersonal regulation) compared to waitlist control in a community sample of Veterans who screened positive for either PTSD and/or depression."		
**1b-ii) Level of human involvement in the METHODS section of the ABSTRACT**		
"Peer-supported mhealth programs hold the promise of providing a low-burden approach to increasing access to care and improving mental health. While peer support has been shown to improve engagement into care, there is limited investigation about the impact of peers on symptom outcome. Trauma-exposed populations frequently suffer from co-occurring posttraumatic stress and depressive symptoms as well as difficulties in day-to-day functioning. This study evaluated the potential benefits of a peer-supported transdiagnostic mhealth program on symptom outcomes and functioning."		
**1b-iii) Open vs. closed, web-based (self-assessment) vs. face-to-face assessments in the METHODS section of the ABSTRACT**		
"A total of 178 eligible Veterans were enrolled in the study using a 2:1 randomization scheme with 117 assigned to BPS webSTAIR and 61 assigned to the waitlist control. PTSD and depressive symptoms as well as emotion regulation, and psychosocial functioning were assessed via phone at pretreatment, posttreatment, and 8-week follow-up. Mixed-effects models were used to assess change in outcome measures across timepoints and evaluate the impact of module completion on outcomes. Exploratory analyses were conducted to determine whether number and type of peer interactions influenced outcomes."		
**1b-iv) RESULTS section in abstract must contain use data**		
"Participants randomized to BPS webSTAIR reported significantly greater improvement on all outcome measures at posttreatment compared to the waitlist control (d=-0.48 to -0.64), and gains were maintained at 8-week follow-up. Those who completed more modules reported greater improvement on all outcomes (d=-0.64 to -0.83). An initial cohort of participants who were required to chat with a peer coach to progress exchanged more messages per module but were less likely to complete the program, compared to a later cohort for whom chatting was optional."		
**1b-v) CONCLUSIONS/DISCUSSION in abstract for negative trials**		
"BPS webSTAIR was effective in improving PTSD and depression symptoms, emotion regulation, and psychosocial functioning in Veterans. Peer-supported transdiagnostic mhealth programs may be a particularly efficient, effective and low-burden approach to improving mental health among trauma-exposed populations. Future research should evaluate how best to integrate peer support in mhealth programs to optimize access to care and treatment outcomes."		
INTRODUCTION		
**2a-i) Problem and the type of system/solution**		

"Given the evidence supporting the success of brief, peer-supported STAIR in reducing a range of symptoms, along with growing support for the effectiveness of web-based mental health programs, a six-session peer-supported version of webSTAIR was developed. This abbreviated version of the webSTAIR protocol can be completed in a shorter time frame than the 10-session webSTAIR and integrates support from trained Veteran peers at the end of each module. The potential benefits of this program, if successful, is the availability of a low-burden, brief mhealth program that is effective in reducing PTSD and depression symptoms as well as in improving emotion regulation and psychosocial functioning."		
**2a-ii) Scientific background, rationale: What is known about the (type of) system**		
"Literature reviews have indicated that the presence of peers increases engagement into treatment across individuals with a variety mental health difficulties and disorders, treatment modalities and delivery strategies [21,22]. In particular, studies assessing the use of peer-supported mhealth among trauma-exposed Veterans have demonstrated increased engagement into treatment [23]. However, randomized controlled trials assessing the impact of peers on symptom change are relatively few in number [24] including for mhealth interventions [25]."		
**Does your paper address CONSORT subitem 2b?**		
"The aim of this study was to assess the benefits of BPS webSTAIR compared to a Waitlist control condition among U.S. Veterans with symptoms of PTSD, depression, or both. We hypothesized that participants assigned to the BPS webSTAIR condition would experience significantly greater improvement in PTSD symptoms and depressive symptoms (primary outcomes) as well as in emotion regulation, and psychosocial functioning (secondary outcomes) compared to Waitlist, and that these gains would be maintained at an 8-week follow up. Exploratory analyses were conducted to determine whether number and type of peer interactions influenced outcomes. The study was funded by the National Center for PTSD Dissemination and Training Division within the VA Palo Alto Health Care System."		
METHODS		
**3a) CONSORT: Description of trial design (such as parallel, factorial) including allocation ratio**		
"Randomization was allocated at a 2:1 ratio (webSTAIR to Waitlist) and was computer-generated via an algorithm developed by staff otherwise not involved in the study. Participant randomization assignment was indicated upon opening the program url. Participants in the BPS webSTAIR condition received instructions to start the program within 2 weeks following the assessment and to complete the 6-module program within 10 weeks."		
**3b) CONSORT: Important changes to methods after trial commencement (such as eligibility criteria), with reasons**		
Not applicable - no changes to methods were needed.		
**3b-i) Bug fixes, Downtimes, Content Changes**		
Not applicable - no Bug fixes, Downtimes, Content Changes were needed.		
**4a) CONSORT: Eligibility criteria for participants**		
"Individuals were eligible if they were a Veteran or military personnel, over twenty-one years of age, endorsed experiencing at least one traumatic event, reported symptoms of PTSD or depression, or both, as indicated by a score of ≥3 on the Primary Care PTSD screen for DSM-5 (PC-PTSD-5) [39] and/or ≥2 the Patient Health Questionnaire-2 items (PHQ-2) [40], were able to read and write in English and had a PC computer and stable internet connection. Exclusion criteria were determined during the phone assessment and were (1) the presence of significant suicidality as indicated by the presence of a plan and means, (2) the presence of cognitive difficulties or active psychosis that would indicate low likelihood of benefiting from treatment, and (3) current participation in trauma-focused treatment."		
**4a-i) Computer / Internet literacy**		
"(2) the presence of cognitive difficulties or active psychosis that would indicate low likelihood of benefiting from treatment"		
**4a-ii) Open vs. closed, web-based vs. face-to-face assessments:**		
"Candidates for study participation were recruited via social media advertisements and directed to complete a short set of online screening questions."		
**4a-iii) Information giving during recruitment**		
"Following the screening, study candidates were contacted via phone and scheduled for a 30-60-minute phone assessment with the study coordinator to assess inclusion and exclusion criteria, receive an explanation of the program and complete (verbal) informed consent."		
**4b) CONSORT: Settings and locations where the data were collected**		
"Data were collected online and stored in a secure VA approved environment."		
**4b-i) Report if outcomes were (self-)assessed through online questionnaires**		
"Data were collected online and stored in a secure VA approved environment."		
**4b-ii) Report how institutional affiliations are displayed**		
We did not include institutional affiliations in our advertisements.		
**5) CONSORT: Describe the interventions for each group with sufficient details to allow replication, including how and when they were actually administered**		
**5-i) Mention names, credential, affiliations of the developers, sponsors, and owners**		

There is no conflict of interest.		
**5-ii) Describe the history/development process**		
"The program evaluated, webSTAIR, is a web-based program adapted from Skills Training in Affective and Interpersonal Regulation (STAIR), a manualized evidence-supported cognitive behavioral intervention for trauma-exposed individuals with symptoms of PTSD and/or depression that focuses on improving psychosocial functioning by building emotion regulation and interpersonal skills. Investigations of 10-12 session STAIR have reported large effect sizes in the reduction of PTSD and depression as well as in emotion regulation and psychosocial impairment (Cloitre et al., 2023; [32]. Similar outcomes have been obtained in a briefer 6-session version, including in a randomized controlled trial in primary care [33] and in an open trial delivered by peers in a low-income primary care community service [34]. In parallel to the STAIR studies, investigations of webSTAIR assessed a 10-module version with weekly coaching sessions from licensed mental health providers. These studies have included two open trials [35, 36] and one comparison trial [37]. The open trials found significant improvements with moderate to large effect sizes for PTSD, depression, emotion regulation difficulties and psychosocial impairment and the comparison study, with a noninferiority design, found that reducing coaching support to biweekly (5 coaching sessions) from weekly (10 coaching sessions) resulted in noninferior benefits on all outcomes."		
**5-iii) Revisions and updating**		
**5-iv) Quality assurance methods**		
**5-v) Ensure replicability by publishing the source code, and/or providing screenshots/screen-capture video, and/or providing flowcharts of the algorithms used**		
**5-vi) Digital preservation**		
**5-vii) Access**		
"If eligible for the study, the participants were provided access to the program. Randomization was allocated at a 2:1 ratio (webSTAIR to Waitlist) and was computer-generated via an algorithm developed by staff otherwise not involved in the study. Participant randomization assignment was indicated upon opening the program url. Participants in the BPS webSTAIR condition received instructions to start the program within 2 weeks following the assessment and to complete the 6-module program within 10 weeks. Assessments were conducted via phone with study staff at pretreatment, posttreatment (10-week mark), and an eight-week follow-up. Participants in the Waitlist condition were given the information that they would have access to the webSTAIR program in 10 weeks and completed assessments at pretreatment and posttreatment (10-week mark). Waitlist participants were then given access to webSTAIR. Participants were paid 40 dollars for the pretreatment assessment and 60 dollars for the post and the follow-up assessments."		
**5-viii) Mode of delivery, features/functionalities/components of the intervention and comparator, and the theoretical framework**		
"The BPS webSTAIR intervention is a shortened, six-module version of webSTAIR adapted from brief STAIR [33]. The six modules of BPS webSTAIR focus on emotional awareness, emotion management through the body, thought, and behavior channels, distress tolerance, and self-compassion. The program integrates text, audio, and video delivery of psychoeducation with interactive exercises and worksheets to help patients understand and internalize the concepts introduced. At the end of each module, participants were prompted to complete a chat session with a Veteran peer coach to help clarify program content and answer any questions they might have about how to implement the skills presented in the module. Interaction with the peer occurred via secure instant messaging on the program platform."		
**5-ix) Describe use parameters**		
"Participants in the BPS webSTAIR condition received instructions to start the program within 2 weeks following the assessment and to complete the 6-module program within 10 weeks. Assessments were conducted via phone with study staff at pretreatment, posttreatment (10-week mark), and an eight-week follow-up. Participants in the Waitlist condition were given the information that they would have access to the webSTAIR program in 10 weeks and completed assessments at pretreatment and posttreatment (10-week mark). Waitlist participants were then given access to webSTAIR."		
**5-x) Clarify the level of human involvement**		
"At the end of each module, participants were prompted to complete a chat session with a Veteran peer coach to help clarify program content and answer any questions they might have about how to implement the skills presented in the module. Interaction with the peer occurred via secure instant messaging on the program platform. Participants could message peers as often as they liked while they were on the platform."		
**5-xi) Report any prompts/reminders used**		
"... participants were also able to contact the research coordinator by phone or text message for technical and program support."		
**5-xii) Describe any co-interventions (incl. training/support)**		
"Exclusion criteria were determined during the phone assessment and were... current participation in trauma-focused treatment."		
**6a) CONSORT: Completely defined pre-specified primary and secondary outcome measures, including how and when they were assessed**		

"We hypothesized that participants assigned to the BPS webSTAIR condition would experience significantly greater improvement in PTSD symptoms and depressive symptoms (primary outcomes) as well as in emotion regulation, and psychosocial functioning (secondary outcomes) compared to Waitlist, and that these gains would be maintained at an 8-week follow up."		
**6a-i) Online questionnaires: describe if they were validated for online use and apply CHERRIES items to describe how the questionnaires were designed/deployed**		
**6a-ii) Describe whether and how "use" (including intensity of use/dosage) was defined/measured/monitored**		
"Metadata on chat sessions, including the number of messages exchanged with a peer coach, were collected at the participant level. Measures of program engagement within the BPS webSTAIR condition included number of modules completed, total number of messages exchanged, and average number of messages exchanged per module."		
**6a-iii) Describe whether, how, and when qualitative feedback from participants was obtained**		
**6b) CONSORT: Any changes to trial outcomes after the trial commenced, with reasons**		
"Data were collected online and stored in a secure VA approved environment."		
**7a) CONSORT: How sample size was determined**		
**7a-i) Describe whether and how expected attrition was taken into account when calculating the sample size**		
**7b) CONSORT: When applicable, explanation of any interim analyses and stopping guidelines**		
"We hypothesized that participants assigned to the BPS webSTAIR condition would experience significantly greater improvement in PTSD symptoms and depressive symptoms (primary outcomes) as well as in emotion regulation, and psychosocial functioning (secondary outcomes) compared to Waitlist, and that these gains would be maintained at an 8-week follow up."		
**8a) CONSORT: Method used to generate the random allocation sequence**		
"Randomization was allocated at a 2:1 ratio (webSTAIR to Waitlist) in blocks of 10, and was computer-generated via an algorithm developed by staff otherwise not involved in the study... A total of 178 eligible Veterans were enrolled in the study using a 2:1 randomization scheme with 117 assigned to BPS webSTAIR and 61 assigned to the waitlist control."		
**8b) CONSORT: Type of randomisation; details of any restriction (such as blocking and block size)**		
"Randomization was allocated at a 2:1 ratio (webSTAIR to Waitlist) in blocks of 10, and was computer-generated via an algorithm developed by staff otherwise not involved in the study."		
**9) CONSORT: Mechanism used to implement the random allocation sequence (such as sequentially numbered containers), describing any steps taken to conceal the sequence until interventions were assigned**		
"Randomization was allocated at a 2:1 ratio (webSTAIR to Waitlist) in blocks of 10"		
**10) CONSORT: Who generated the random allocation sequence, who enrolled participants, and who assigned participants to interventions**		
"Randomization... was computer-generated via an algorithm developed by staff otherwise not involved in the study."		
**11a) CONSORT: Blinding - If done, who was blinded after assignment to interventions (for example, participants, care providers, those assessing outcomes) and how**		
**11a-i) Specify who was blinded, and who wasn't**		
"Assessments for posttreatment (10-week mark) and an eight-week follow-up were conducted via phone by study staff who were unaware of the condition and assessment period."		
**11a-ii) Discuss e.g., whether participants knew which intervention was the "intervention of interest" and which one was the "comparator"**		
**11b) CONSORT: If relevant, description of the similarity of interventions**		
Not applicable to our design.		
**12a) CONSORT: Statistical methods used to compare groups for primary and secondary outcomes**		
"Analyses were conducted using R (Version 4.2.2)... all analyses were conducted using the intention-to-treat sample of randomized participants... For the main analyses on our primary and secondary outcomes, linear mixed-effects models were fitted using restricted maximum likelihood (REML) estimation with the lmer function in the package lme4."		
**12a-i) Imputation techniques to deal with attrition / missing values**		

"Missing data was addressed using multiple imputation. Joint multiple imputation of missing values at posttreatment and follow-up assessments was carried out using the panImpute function in package mitml. Due to some of the presently reported analyses being specific to webSTAIR participants, while other analyses were relevant to the full sample, multiple imputation procedures were carried out separately for these two contexts to create two sets of imputed data. The between-group imputed datasets consisted of baseline and posttreatment data for all participants, whereas the within-group imputed datasets included baseline, posttreatment, and follow-up data for only the participants randomized to webSTAIR. In each case, 100 data sets were generated with missing values imputed. Prior to imputing the data, chi square tests and t tests were used to identify predictors of missingness and baseline characteristics related to non-missing values on outcome variables. The missing data correlates identified from these preliminary analyses (employment status, education level, service era, and gender) were included as auxiliary variables in the imputation models. To ensure that any interactions between time and condition were appropriately preserved in the between-group imputed datasets, this interaction term was included as a predictor in the between-group imputation model. Results were pooled across imputed data sets using the testEstimates function in the mitml package."		
**12b) CONSORT: Methods for additional analyses, such as subgroup analyses and adjusted analyses**		
"To evaluate the impact of program completion on outcomes, module completion was categorized as 'None' (did not complete any online content, including the Welcome Module), 'Some' (completed the Welcome Module and no more than Module 2), or 'Moderate to Complete' (completed Module 3 or higher). Within each completion group, linear mixed-effects models were constructed for each outcome measure to evaluate within-group change across time."		
RESULTS		
**13a) CONSORT: For each group, the numbers of participants who were randomly assigned, received intended treatment, and were analysed for the primary outcome**		
"A total of 178 eligible Veterans were enrolled in the study using a 2:1 randomization scheme with 117 assigned to BPS webSTAIR and 61 assigned to the waitlist control."		
**13b) CONSORT: For each group, losses and exclusions after randomisation, together with reasons**		
"A total of 178 eligible Veterans were enrolled in the study using a 2:1 randomization scheme with 117 assigned to BPS webSTAIR and 61 assigned to the waitlist control. As indicated in the CONSORT chart (Figure 1) 201 participants were initially enrolled but 21 participants (14 in webSTAIR and 7 in waitlist) were identified as ineligible for the study predominantly due to not being U.S. citizens/veterans and were removed from the study."		
**13b-i) Attrition diagram**		
"As indicated in the CONSORT chart (Figure 1) 201 participants were initially enrolled but 21 participants (14 in webSTAIR and 7 in waitlist) were identified as ineligible for the study predominantly due to not being U.S. citizens/veterans and were removed from the study."		
**14a) CONSORT: Dates defining the periods of recruitment and follow-up**		
"Recruitment began 10/16/2020 and follow-up ended 9/28/2022."		
**14a-i) Indicate if critical "secular events" fell into the study period**		
**14b) CONSORT: Why the trial ended or was stopped (early)**		
Not applicable to study. "Recruitment began 10/16/2020 and follow-up ended 9/28/2022."		
**15) CONSORT: A table showing baseline demographic and clinical characteristics for each group**		
"Sample sociodemographic characteristics are shown in Table 1... Nearly 58 percent were approved for VA service connection status for PTSD (at any disability rating), 27.0 had never applied, 8.4 had applied or had an application under review, and 6.7 had an application denied. A total of 32.0 percent were currently engaged in other (non-trauma focused) counseling, and 6.7 percent had received evidence-based treatment for PTSD or related conditions within the past year. No significant differences by condition were found for any sociodemographic item. At pretreatment, the two conditions did not significantly differ on any outcome measures except for the PHQ-8."		
**15-i) Report demographics associated with digital divide issues**		
"Similar to other Veteran samples, most participants were male. Roughly two-thirds were White or Caucasian, and the average age was 48.08 (SD = 9.04)."		
**16a) CONSORT: For each group, number of participants (denominator) included in each analysis and whether the analysis was by original assigned groups**		
**16-i) Report multiple "denominators" and provide definitions**		
"Table 3 shows the estimated coefficients and effect sizes for pre- to posttreatment change in the outcome measures by module completion group in the BPS webSTAIR condition. Participants who completed no modules (n = 25; coded as 'None') did not experience a significant change in PCL-5 score from pretreatment to posttreatment (Est. = -1.88; SE = 4.72; P = 0.69; d = -0.13). Those who completed at least the Welcome Module but did not move beyond the second full module of BPS webSTAIR (n= 48; coded as 'Some') experienced a mean PCL-5 total score reduction of 6.77 (SE = 3.16; P = .03; d = -0.50). Participants who completed three or more (out of six total) modules of BPS webSTAIR (n= 44; coded as 'Moderate to Complete'), experienced an estimated PCL-5 total score reduction of 10.13 points from pretreatment to posttreatment (SE = 2.49; P < .001; d = -0.64)."		
**16-ii) Primary analysis should be intent-to-treat**		

"Consequently, all analyses were conducted using the intention-to-treat sample of randomized participants."		
**17a) CONSORT: For each primary and secondary outcome, results for each group, and the estimated effect size and its precision (such as 95% confidence interval)**		
Estimated effect sizes, P-values, and 95% confidence intervals are presented for all outcomes in Tables 2-3.		
**17a-i) Presentation of process outcomes such as metrics of use and intensity of use**		
Results of statistical analyses of attrition and intensity of use are presented in Tables 3-4.		
**17b) CONSORT: For binary outcomes, presentation of both absolute and relative effect sizes is recommended**		
Not applicable, since there are no binary outcomes.		
**18) CONSORT: Results of any other analyses performed, including subgroup analyses and adjusted analyses, distinguishing pre-specified from exploratory**		
Data from specific cohorts are presented in Table 4.		
**18-i) Subgroup analysis of comparing only users**		
Table 3 presents data on outcomes by groups based on level of completion.		
**19) CONSORT: All important harms or unintended effects in each group**		
None were reported in this study.		
**19-i) Include privacy breaches, technical problems**		
None were reported in this study.		
**19-ii) Include qualitative feedback from participants or observations from staff/researchers**		
DISCUSSION		
**20) CONSORT: Trial limitations, addressing sources of potential bias, imprecision, multiplicity of analyses**		
**20-i) Typical limitations in ehealth trials**		
"The present study has limitations. First, we relied fully on self-report measures administered by phone. Second, for ethical reasons, we did not maintain the waitlist group during the follow-up period and so were unable to conduct between-group analyses of change from pretreatment to follow-up. Third, there were limitations regarding the opportunity to evaluate the role of peer coaching. Because the requirement to participate in chat sessions differed between cohorts, it was not possible to combine the three cohorts to assess their impact on outcome in a meaningful way. Furthermore, chat transcripts were not accessible and thus we were limited in our ability to explain why more chat messages were not associated with better outcomes. It could be that the conversations were routine and pragmatic (e.g., resolving technical issues) versus meaningful guidance about the content of webSTAIR."		
**21) CONSORT: Generalisability (external validity, applicability) of the trial findings**		
**21-i) Generalizability to other populations**		
"This study adds to existing literature on mhealth by demonstrating that a brief, transdiagnostic program, supported by peers rather than therapists, can provide moderate to large improvements in PTSD and depression symptoms as well as in emotion regulation and psychosocial functioning."		
**21-ii) Discuss if there were elements in the RCT that would be different in a routine application setting**		
**22) CONSORT: Interpretation consistent with results, balancing benefits and harms, and considering other relevant evidence**		
**22-i) Restate study questions and summarize the answers suggested by the data, starting with primary outcomes and process outcomes (use)**		
"The results of the study indicated that as compared to Waitlist, webSTAIR supported by peers is effective in improving symptoms of PTSD and depression, as well as emotion regulation and psychosocial functioning, with gains maintained at 8-week follow-up. The within-group pre to post effect sizes for the webSTAIR participants ranged from 0.48 to 0.64, somewhat larger than those found for other peer-supported mhealth programs [21, 22]. Brief Peer-Supported (BPS) webSTAIR offers a convenient and potentially more scalable option for Veterans who have limited time available, lack access to a mental health provider, or prefer to work through the content in a self-guided manner."		
**22-ii) Highlight unanswered new questions, suggest future research**		
"It would be valuable to identify baseline characteristics of clients who might substantially benefit from peer support versus those who will not. In addition, it may be that clients who will be helped by peer support will experience greater benefit if the peer was proactive in reaching out to the client. In qualitative interviews, mental health coaches have pointed to the value of building in proactive contact initiated by coaches rather than placing the onus on participants to engage [51]. In this study the peers were available to the veterans to chat, but the veterans initiated the contact. Future efforts to enhance the impact of peers may involve modification regarding how proactive they are."		
Other information		
**23) CONSORT: Registration number and name of trial registry**		

ClinicalTrials.gov NCT04286165		
**24) CONSORT: Where the full trial protocol can be accessed, if available**		
ClinicalTrials.gov NCT04286165		
**25) CONSORT: Sources of funding and other support (such as supply of drugs), role of funders**		
VA National Center for PTSD.		
**X26-i) Comment on ethics committee approval**		
"The study was approved by the IRB of the university affiliated with the VA."		
**x26-ii) Outline informed consent procedures**		
"Following the screening, study candidates were contacted via phone and scheduled for a 30-60-minute phone assessment with the study coordinator to assess inclusion and exclusion criteria, receive an explanation of the program and complete (verbal) informed consent."		
**X26-iii) Safety and security procedures**		
The study was approved by the IRB of the university affiliated with the VA... Data were collected online and stored in a secure VA-approved environment."		
**X27-i) State the relation of the study team towards the system being evaluated**		
There are no conflicts of interest.		
